# Ayurvedic protocols of chronic pain management: spatiotemporality as present moment awareness and embodied time

**DOI:** 10.3389/fpain.2024.1327393

**Published:** 2024-01-26

**Authors:** Vinita Agarwal

**Affiliations:** Department of Communication, Salisbury University, Salisbury, MD, United States

**Keywords:** mind–body approaches, complementary and integrative medicine, environment, chronic pain management, Ayurveda, ecological, spatiotemporality, embodied time

## Abstract

**Background:**

Temporality is understood as the subjective perception of the flow of chronological time and is a central component of contemporary and integrative medicine approaches. Although temporal dynamics are recognized as central to the processes associated with chronic pain (CP), the temporal management of CP is inadequately understood in pain research.

**Research question:**

How is temporality conceptualized in Ayurvedic protocols of CP management?.

**Method:**

Ayurvedic physicians (*N* = 10) from India were recruited through purposive and snowball sampling. A semi-structured interview protocol was employed to gather qualitative data focusing on the Ayurvedic protocol employed to treat CP patients. The interviews were audio-recorded, professionally transcribed, and thematically analyzed. Member validation, participant voice, and researcher self-awareness were employed to strengthen reliability and validity.

**Findings:**

An ontologically grounded thematic exploration of Ayurvedic protocols illustrates that temporality is conceptualized in CP as spatiotemporal present moment awareness (PMA) and embodied time (ET). Spatiotemporality as PMA references an awareness of the relationality of cognitive temporal movement, *dosha* operations, and their pathophysiological expression in the body. Spatiotemporality as ET is conceptualized as awareness of the expression of time in embodied emotional and psychosocial processes as in the movement of the breath through the body, the movement of body sensations over time, and in their intersection with consciousness.

**Discussion:**

The study findings present an experiential and relational framework situating spatiotemporality ontologically as an organizing principle in CP management. While temporality focuses on the representation of experiences and relations over time, spatiotemporality foregrounds a constructionist approach by centering the embodied spatial cognitive expression of time, consciousness, and subjective experience.

## Introduction

Chronic pain (CP) is the leading indication for the use of complementary and integrative medicine (CIM) approaches (1). Approximately 33% of adults and 12% of children in the United States use CIM for a range of conditions such as the management of back, neck, and joint pain (2–5). CIM therapies support enhanced ownership of care and shared decision-making (6–8) and employ diverse ontological frameworks to emphasize an experiential and relational praxis (9–14). Studies focusing on body ownership in CP management (15) emphasize the individualized nature of pain to underscore the benefits provided by multisensory integration and self-awareness from CIM therapies employing mindfulness, deep breathing, gratitude, and relaxation techniques (9, 16–19). CIM therapies employ temporality (in movement, mindfulness, and seasonality) in targeting subjective wellbeing and health-related quality of life (20) through centering the organization of experiences and relations over time. Temporality emphasizes the subjective perception of the flow of chronological time through the past, present, and future, as experienced through historicity in events and movement, or understood phenomenologically as simultaneity, subjective duration, successiveness, and the subjective present. CIM therapies involving meditation and mindfulness focus on awareness of the passage of time and self-consciousness, distinguishing between self-time perspective (internal time), world duration (external time), and the embodied self as associated with the subjective experience of internal time (21). The consciousness of subjective time is experienced in relationship with the temporality of pain experience through being conscious of the present as a moment in time (22). Temporality in CP can be experienced as a form of depersonalization separating the field of presence from the past and the future through embodied functions such as implicit bodily memory and bodily anticipation (23). Although such findings address temporality, the embodied self, and subjective time in CP, there is an inadequate understanding of how temporality is conceptualized by CIM providers and how CIM providers employ temporality in managing CP. Similarly, the patient's subjective experience of pain temporality in CP management also remains underexamined (24). The pain experience disrupts lived temporality, suggesting a need to examine how time is experienced in the CP phenomenon (25).

The multicomponent nature of temporality is underscored in examinations of how time (e.g., through time perception) contributes to the construction of health and flourishing. Phenomenologists have attended to the construction of temporality through the temporally contracted disease progression model to a focus on the present discrete snapshot of disease in the diagnosis and prognosis process (26). In humans, time perception encompasses the integrating and evaluating temporal facet of memories, emotions, and experiences. It has been examined through constructs such as present fatalism, morning–evening orientation (27), present-hedonism orientation, and subjective and objective passage of time to understand the interconnections between circadian typology, individual time perception, and passage of time (28). Interest in time perception has spanned identification of its neurobiological basis through a focus on brain structure variations (28) to understand its relationship with the flow of time (29), image of time (30), and neural bases for perception of time (31) to understanding its role in behavioral health through explicating its associations with self-regulation (32), anxiety, and depression (33), among other outcomes.

More recently, consciousness and self-reflexivity have been recognized as components of temporality in the pain experience. Investigations of neural correlates of mental phenomena such as the self, consciousness, and perception have examined spatiotemporality as a mechanism for understanding how the brain's activity in constructing inner time and space is manifested in cognition and mental features (34). The neurophysiological estimation of time and the perception of self are considered as sharing a common neural substrate, suggesting that alongside bodily arousal and attentional capture, self-reflexivity may also be a component of dilated subjective time during the experience of pain (35). Imaging studies suggest a spatiotemporal reorganization of brain activity and CP cognition whereby conscious experiencing of unpleasant sensory or emotional sensations through cognitive processing is perceived as pain (36). One way of considering temporality, thus, is as embedded in the neurophysiological expression of CP. The inter-individual difference in the neurophysiological encoding of painful stimuli and memory emphasizes how the anxiety influences the handling of the noxious vs. the innocuous stimuli (36).

A focus on anxiety and sensory intensification in the pain experience emphasizes pain development as constituting its own time within time, as through slower life routines, greater uncertainty, and a limited future time perspective. Temporality has been assessed in chronic (or persistent) pain measures through pain temporal pattern, pain duration, and future time perspective. Future time perspective is understood as the subjective perception of time as limited or expansive in an assessment of future opportunities and the amount of time one has left to live (37). In contrast, flow experiences are temporally grounded in their perception of life in its entirety within a unified flow process and with a unified sense of purpose (38). Some studies have examined how pain limits individuals' qualitative perceptions of the future by challenging their ability to construct flow experiences that envision a future or by negatively affecting their future outlook (39). Moreover, a limited future time perspective is associated with greater pain-related activity interference whereas longer pain duration as in CP has been associated with a more expansive future time perspective (40).

Along with future time perspective and flow experiences, the conceptualization of temporality in CP has focused on balanced time perspective or an adaptive engagement with past, present, and future time perspectives in alignment with contextual elements (41). The balanced time perspective is associated with myriad positive psychosocial outcomes including higher life satisfaction (42), emotional intelligence (43), psychological need satisfaction and gratitude (44), happiness (45), attributional complexity, wisdom, and mental health (46), and mindfulness (47, 48). Temporally, mindfulness has been understood as a non-judging and open way of relating toward the present moment (49) associated with constructs such as knowledge of one's true self (50), self-awareness, and meaning in life (51). Balanced time perspective is also associated with outcomes such as emotional regulation and affect (52); flow, mindfulness, and mental health (53); and adaptive identity styles and flourishing (46). Because cognitive and emotional processes involved in the construct meaning-in-life draw upon distinct temporal frames, the meaning-making and time perspective relationship can be modified in illness domains such as CP. Similar to mindfulness, the construct meaning in life is also temporally grounded, referencing an orientation to the world that embraces the past, present, and future (54, 55). For instance, meaning-making through the past orientation has been associated with autobiographical reflections (56), in the present by staying in the moment or present-focused with mindfulness (33), and in its future orientation with wellbeing processes (57). The association of time perception (e.g., as future and balanced time perspective) with subjective wellbeing, meaning-making, and mental health (including positive associations with love, joy, life satisfaction, wisdom, growth narratives, gratitude, life satisfaction, and flow experiences) suggests how understanding temporality is central to managing anxiety, depression, and negative affect associated with CP (58, 59).

Although temporal dynamics are central to the pathophysiological, psychosocial, and behavioral processes (including mindfulness, meaning in life, and flourishing) associated with CP and its clinical management, their association with the temporal facets of the pain experience (e.g., frequency, duration, and intensity of pain episodes) has not received adequate attention in pain research (60). Methodological approaches such as ecological momentary assessments have examined the modulation of pain experiences and their dynamic nature over time with patients' natural daily environments, variability in context and activity dependence, and diurnal cyclicity. However, the temporality of these associations has not been sufficiently examined in CP management [e.g., circadian variability of CP in rheumatoid arthritis (61–64)]. CIM approaches such as the Ayurvedic system of medicine focus on temporality (e.g., in the characterization of *dosha* dominance in the lifespan, diurnally, seasonally, and ecosystemically; where *dosha*s reference the manifestation of the three forms of energy, *vata, pitta*, and *kapha*, that govern the operations of the body) and provide an ontologically distinct medical model for understanding temporality in CP management. For instance, Ayurvedic medical protocols of CP categorize musculoskeletal pain conditions as dominated by *vata* and *kapha doshas* and as affected by diurnal (morning stiffness and increased pain intensity in rheumatoid arthritis are seen as an instance of clinical presentation in musculoskeletal pain) and seasonal changes (65). Ayurvedic treatments (*Amavata* for diagnosis and *virechanakarma,* including *swedana* in the morning) prioritize the observation of temporality in the diagnosis, prognosis, and therapeutic processes [e.g., by avoiding daytime sleep, *divaswapna* (66)]. Thus, an examination of Ayurvedic conceptualization of temporality can further conceptual understandings of temporality and provide innovative insights into CP management from distinctive ontological medical models. This study examines how temporality is conceptualized in Ayurvedic protocols of CP management through an in-depth thematic analysis of semi-structured interviews with Ayurvedic physicians.

## Materials and methods

### Participants and procedures

The study analytic procedures have been described elsewhere in detail (12). The goal of this concise, exploratory qualitative study is to provide deeper insights into the phenomenon of interest (CP management in Ayurvedic protocols). To achieve this goal, a methodological approach centered on expert sampling of Ayurvedic physicians in India (*N *= 10) with a Bachelors of Ayurvedic Medical Science (BAMS) degree was employed to gather information-rich data with limited resources (see Table 1 for participant description) (67). The study participants were recruited from a city in the southwest and one from the northwest region of India from a professional training center and based on public practitioner searches. Participant recruitment was conducted following the criterion of maximum variation, availability, willingness to participate, and ability of participants to communicate experiences and opinions in English in an articulate manner (68). The study’s participant recruitment was concluded once a representative depth and breadth of perspectives was obtained from a small yet tightly focused pool based on saturation within a specific content domain and where participant responses showed low variability and high homogeneity (69). A semi-structured in-depth interviewing protocol (Appendix 1) was employed with a combination of open-ended domain-level questions and probes for the exploration of participant beliefs, thoughts, and practices. The case study method was consulted to elucidate the conceptual dimensions of an underexamined, niche conceptual domain and to identify similarities and differences in the phenomenon of interest (68, 70). To gain an understanding of CP management approaches in-context, the researcher incorporated observations of Ayurvedic provider–patient interactions, artifacts, offices, and hospitals *in situ* in national and regional urban centers and through official tours of national and international Ayurvedic medical institutions in India (Table 2 provides a summary of the research methodology flow). Participant interviews were conducted in practice sites, residences, and hospitals and were audio-recorded, professionally transcribed verbatim, and analyzed using thematic analysis (71). The interviews were conducted in English; thus, the participants' occasional recitation of native language verses (e.g., Sanskrit) from original ancient texts such as the *Charaka Samhita* in their interviews was not transcribed. The researcher has native or bilingual proficiency in English and Hindi and elementary proficiency in Sanskrit.

**Table 1:** Participant demographics.

Pseudonym	Age (years)	Education	Specialty	Profession*	Employment (years)***	City†
A	32	MD	*Dravyaguna*	Ayurvedic Physician & Practitioner	9	Pune
		Ayurveda				
		(BAMS)				
B	45	MA, Ayurveda		Ayurvedic physician	20	Pune
		MA,				
		Sanskrit				
C	44	BAMS & MD, Ayurveda	*Kayachikitsa*	Ayurvedic physician	18	Pune
D	51	BAMS	Medicine & Surgery	Ayurveda physician/practitioner	27	Pune/Mumbai
		Ayurveda				
		MS in Ayurvedic Dietetics				
E	33	BAMS	Medicine & Surgery	Ayurveda physician/practitioner	10	Mumbai
		Ayurveda				
F	26	BAMS	Medicine & Surgery	Ayurveda physician/practitioner	2	Delhi
		Ayurveda				
G	34	MD, Ayurveda	Charak Samhita	Ayurveda physician and academician	17	Pune
H	46	BAMS	Medicine & Surgery	Ayurveda Acharya** (Physician)	20	Pune
		Ayurveda				
I	46	BAMS	Medicine & Surgery, Yoga	Ayurveda Consultant, Physician, Yoga teacher	22	Pune
		Ayurveda, MA, Yoga				
J	69	MD, PhD, Ayurveda	Medicine & Surgery	Professor & Government of India	35	Delhi

* Profession as self-described by participant.

** Ayurved Acharya is the Hindi translation for Ayurved Physician.

*** Aggregate reported in cases where participants have had multiple concurrent or additional professional roles (e.g., Ayurvedic physician and yoga teacher or academician).

^†^ Location of current practice reported or where participant was based for a major duration.

**Table 2:** Research design and structure considerations (69).

Philosophical Worldview	Selected Qualitative Strategies of Inquiry	Research Methods	Research Design
Social constructive	Participation	Purposively recruited participants using snowball sampling	Qualitative
Phenomenological	Observation (e.g., Ayurvedic physician–patient meetings; preparation of food in traditional Ayurvedic methods; consumption; lifestyle at Ayurvedic physician homes)	Semi-structured interviews	Small sample size
		Videos and photographs	
Multiple meanings	Field visits (e.g., Ayurvedic centers, national government bodies, and major regional hospitals)	Data analysis: inductive theme analysis	Non-generalizable
Socially and historically contingent construction and interpretation	Immersion *in situ* (e.g., meditation retreat and training; physician home visits)	Interpretative, descriptive analysis and presentation	Rich, complex data context and analysis
		Self-reflexive write-up	
		Internal validation	

### Validity and reliability

The study’s trustworthiness was emphasized by strengthening its validity and reliability through the use of triangulation and data synthesis by supplementing the observation of Ayurvedic physician interactions with their patients (*n *= 5 physician–patient session visits). To bolster the subjective and interpretive nature of the data analytic process, internal validity was supported through face validity and multiple forms of empirical observation. These included visits to Ayurvedic physician offices and homes (*n *= 6), observation of the Ayurvedic belief system in its lived socio-cultural context, and data triangulation through researcher employment of multiple experiential modes (72) including completing a 10-day silent Vipassana meditation retreat and an advanced certification in Ayurvedic diet and nutrition principles. Rigor in the analytic process was incorporated through researcher sensitivity to the culturally specific meanings in bolstering the accuracy of interpretation, identification of similar and contradictory themes, and presentation of multiple participant voices (72, 73).

### Ethical considerations

The International Review Board (IRB) approval (Human Subjects Review Committee, FWA00020237) for the study protocol (Protocol # 52) was received on 29 April 2019, for the larger study goal of examining Ayurvedic mind–body therapies in CP management. Informed consent was obtained through oral administration of the informed consent (audio-recorded) prior to participation in the study and receipt of an electronic or hard copy of the informed consent.

### Research design and researcher role

The bottom-up research design employing a small sample size allowed for the exploration and thick (i.e., rich and detailed) description of an underexamined conceptual domain. The research design facilitated flexibility of exploration of the philosophy and practice of an ontologically distinct whole medicine system (Table 2). One limitation of a purposeful sampling strategy is the lack of knowledge of the range of variation at the onset of the study; thus, a combination of conceptual understanding of the research domain coupled with iterative data analysis alongside the data gathering process supported the determination of emergent data saturation (74). A second limitation is the bias stemming from a study designed and conducted by a single researcher with a philosophical background in Ayurvedic lifestyle on the interpretation of the concepts in relationship with biopsychosocial and integrative medicine clinical approaches.

### Case study inductive data analytic procedures

This study reports findings derived from a subset of the data. Inductive qualitative content analysis was employed (75) to derive the categories and themes from the data. Critical conceptual insights were integrated into the interpretive analytic processes comprising the phenomenon of study by including participant voices and reducing data into categories and themes through iterative funneling (76). The data analysis process was initiated alongside data gathering. At the end of each interview, the audio recording was reviewed by the researcher to identify themes and ideas deemed as interesting, iterative, or recurring. These were further explored in successive interviews to ensure their dimensions were fully examined. The present study was analyzed solely by the author; thus, these steps helped mitigate researcher bias and strengthen data analysis. To further help mitigate researcher bias in the data analytic process, the researcher sought to cultivate openness to emerging concepts and themes (77) and undertook multiple passes of the data. The first pass of the transcripts examined the environmental–ecological context with subsequent passes narrowing down to the temporal experientiality of CP, to spatiotemporality and its key thematic dimensions (76). The data analytic procedures sought to go beyond observation, description, and categorization to identification of points of distinction and relatedness with existing conventional knowledge bases through a process of abstraction and constant comparison. In the second pass, the researcher conducted a line-by-line process of open coding with a subsequent pass-through salient data to identify axial codes that comprised the descriptive themes by attending to the “patterns, insights, and concepts” (72) (p. 167) that emerged in the descriptive level (e.g., association of cognitive temporal movement with *dosha*s). Relationships among the categories were finalized by keeping interpretation close to participant descriptions to help strengthen reliability through reader corroboration of the themes. Participant confidentiality was maintained in the presentation of their quotes in the findings. Minor corrections for syntax or missing words were made for readability and are presented in parentheses. The participants are referenced by alphabetical letters in parentheses, e.g., [A] or [C].

The reporting of the findings emphasizes participant voices in the spirit of the interpretive nature of an explorative research inquiry, highlighting the researcher's native knowledge alongside participant interviews (please refer to Appendix 2 for author's note regarding limitations in the interpretation of the study premise and findings for Ayurvedic and integrative medicine clinical practice). As a small qualitative study, the findings enhance understandings of temporality and propose an innovative conceptual framework to further its integration in CP management. Owing to the qualitative case study design with a small sample size, the study findings are exploratory and descriptive in nature and non-generalizable. The findings can be further validated and extended through experimental methodologies to enhance understandings of an inadequately understood conceptual domain in CP management.

## Results

Thematic analyses reveal Ayurvedic protocols for pain management and conceptualize temporality in CP as spatiotemporal present moment awareness (PMA) and embodied time (ET).

### Spatiotemporality as present moment awareness

**Figure 1:**
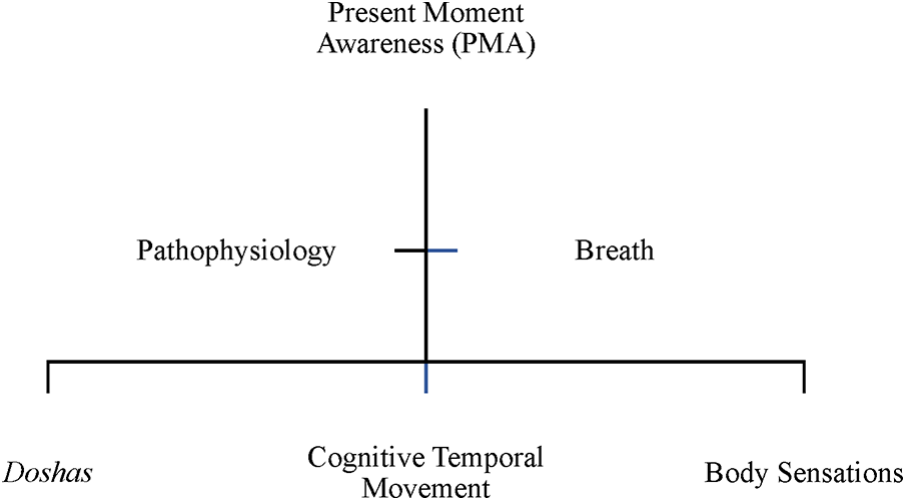
Spatiotemporality as present moment awareness.

**Figure 2:**
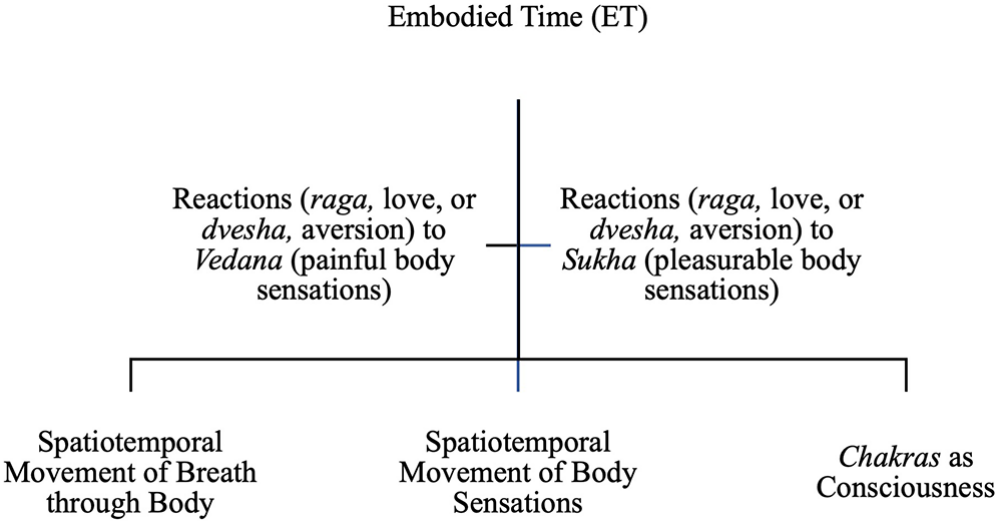
Spatiotemporality as embodied time.

Ayurvedic physicians employ temporality in present moment awareness in CP management by seeking to construct a balance between the spatial–temporal organization of cognitive temporal movement and the *doshas*, among the three *doshas*, and between the cognitive temporal movement, the *doshas*, and *dosha* expression in body sensations (Figure 1). Spatiotemporality is conceptualized in PMA as an awareness of the relationality of cognitive movement, *dosha* operations, and their pathophysiological expression through their movement as thought and intentionality in the body (Figure 2). PMA is conceptualized as the state of awareness in the present regarding cognitive temporal movement with the three body constitution types (*doshas*) and their pathophysiological expressions. Ayurvedic practitioners employ methodological approaches including *mantra* chanting, respiration-breath modulation through yoga and *pranayama* and diet and lifestyle modification to cultivate PMA.

Pain is understood in Ayurvedic medicine as a perception of sensations in the physical, mental, and emotional body. The perception of sensations is an essential component of the *dosha*s in the body. In PMA, the Ayurvedic physician addresses the imbalance of the three *dosha*s with cognitive temporal movements categorized as an unpleasant sensation, or pain. According to H, “whenever you will find pain, there is a role of *vata*,” with there being “three different types of pains.” The form and description of pain is characterized as any form of sensation in the body, whether that be pleasurable or like “needles … shifting … throbbing … sharp.” The spatiotemporal perception of pain indicates the type of *dosha* predominance causing the imbalance. For instance, it can be burning (characteristic of *pitta* imbalance), or dull (characteristic of a *kapha* imbalance) [H]. Accordingly, a spatiotemporal equilibrium or harmony among the *dosha*s, the sense organs, the cognitive system, spiritual system, and the elimination of toxicity, is associated with pleasant sensations characterized by good health and wellbeing. As [H] explained, good health is “when there is harmony among the body, mind, soul, *indriya* (the senses) *dosha*, and *mala* (toxins). If all the body along with its mind and conscious (ness) are working together in harmony then only we can say that the person is in good health.” As [G] explains, “many of the aspects of the body are controlled by the mind through the breath or the *pranayama*”, such as “the relation between v*ata* and the *raja guna* of the mind. *Raja dosha* of the mind … when we control our respiration, we control our mind.” The Ayurvedic physician emphasizes the spatiotemporality of the interrelationships between respiration and the brain and breath and mind. Thought is understood as a cognitive temporal movement, regulated by *vata dosha*: “and when there is a movement, there is a *vata* exercises that control the mind” [G]. The emphasis on movement (e.g., of thought in this instance, and all functions governed by *vata* more generally) underscores the spatiotemporal nature of cognitive processes.

The theme of PMA emphasizes how the Ayurvedic physician looks for a spatiotemporally balanced operation of the *doshas* to create harmony without the dominance of any one *dosha* or accumulation of toxicity (that may be perceived as an unpleasant sensation, pain, or disease). In other words, to understand pain, the Ayurvedic physician starts with the principle that “*mana* (mind) and emotions are very closely related … we need to take care of body and mind while treating the chronic pain management.” One way Ayurveda cultivates PMA is as a cognitive approach to realigning the individual's relationship with pain. One approach in Ayurveda, [H] noted, is “*mantra* chanting or having positive thoughts in your mind … [which] reduces the pain threshold.” Thought and cognitive activity is understood as a form of temporal movement arising from the interaction of the body and mind, referencing the spatiotemporal organizing of the nature of the relations between mind and emotions. *Mantra* chanting focuses positive thoughts centering the body, centering the mind, spirit, and nature relationship in PMA. For the Ayurvedic physician, both the origin and manifestation of diseases are associated with historical time and its relationship with cognitive temporal movement: “whatever disorders take place in mind, they surely affect the body … whatever diseases take place in the body, they certainly affect the mind, so there is a strong interrelation in between mind and body” [B]. Understanding the nature of pain encompasses understanding the “psychological disturbances … profession, stress levels, diet habits” of the patient [H]. Obtaining an understanding of the psychosocial experiences alongside the anatomical, biochemical, and sensory experiences of pain as a sensation allows the Ayurvedic physician to comprehend pain processing spatially through its cognitive temporal movement arising from an imbalance.

To understand CP, [G] examines how pain and sadness in the mind and body mutually influence each other in the patient. The nature of sound is spatiotemporally connected with thoughts (e.g., silent thoughts in the mind or thoughts in an utterance). Thus, sound as employed through *mantras* expresses the cognitive temporal movement of intention. Yoga (combining breath through *pranayama*, body through *asanas*, and mind through meditation and *mantras*), alongside lifestyle and diet modifications, has the function of energizing the mind by strengthening the direction of thoughts that express the movement of negative qualities to positive [G]. The *dosha*s are further modified by their spatiotemporal qualities (or *guna*s). The *gunas* are also targeted in Ayurveda to bring their energy characteristics expressed through sensations in balance for reconceptualizing how the pain sensation is experienced. [G] brings the movement of the body and thoughts in harmony over time through “diet and nutrition, lifestyle management … exercise, yoga *pranayama*, relaxation and breathing techniques, some medicinal herbs, *panchakarma*, and then rejuvenation.” Spatiotemporal analysis of these components at an individual level illustrates the interaction of their energy composition as defined in Ayurveda with the individual's *dosha* balance and working to bring their movement in harmony.

### Spatiotemporality as embodied time

Ayurvedic physicians cultivate embodied time in CP management as an awareness of the expression of time as embodied in the emotional and psychosocial processes of the body. CP management employs ET by cultivating an awareness of spatiotemporality as reflected in the movement of the breath through the body, the movement of body sensations over time, and their intersection with consciousness (Figure 2). To connect body sensations (ranging from painful, *vedana* to pleasant, *sukha*) with consciousness, Ayurvedic practitioners employ approaches such as *pranayama* yoga, diet, herbs, *marma*, *panchakarma*, and *dinacharya* and *ritucharya* (Table 3).

**Table 3:** PMA and ET: balancing and reconceptualizing modalities.

PMA	ET
Balancing and Reconceptualizing Modalities	
*Mantras*	*Pranayama yoga*
Yoga *asanas*	Diet
*Pranayama*	Medicinal herbs
Diet	*Marma*
Medicinal herbs	*Panchakarma*
Meditation	*Dinacharya* and *ritucharya*
*Panchakarma*	
Exercise and lifestyle	

Pain is conceptually understood by the Ayurvedic physician as sensations of both pleasure (*sukha*) and sadness (*vedana*). However, for its treatment as pathology, [A] mentioned that CP is “not directly related with the *atma* (consciousness), means in case of chronic pain … if you go with the Sanskrit shloka, meaning of *vedana* means pain … is essential for your mind, is *sukha*, means pleasantness … The pain who is give very much difficulty to your mind, it's a *dukha*, means sorrowness. So, every condition is related with that definition.” Hence, pain is spatiotemporally embodied sensations expressed over time through the body. The notion of time, or *kaal,* is considered essential in understanding the cause of its expression: “because the pain, how many days pain is occurring? … How many days the things is happening, because … One of the reasons of any disease is *kaal* [time]” [A]. Spatiotemporality embodies the expression of time through the body as sensations of pain and pleasure.

ET is expressed in the spatiotemporal movement of the breath through the body. According to [H], “*pranayama* yoga or relaxation techniques … increase the *prana* or energy force in your body. Also, they remove the obstructions in the body and does the help to reduce the pain threshold.” The obstructions (or blocks) can be psychosocial or pathophysiological. Through its spatiotemporal movement, breath modulates and aligns thought and the internal functions of the body. In fact, “emotionally *mana* [mind] is in the heart and the brain is the functional part of *mana* [as consciousness ]… herbs like *brahmi*, *ashwagandha*, then *vata*, *yashtimadhu* *…* reduce the mental pressure and ultimately the pain” by targeting the mental functions. Thus, the spatiotemporal movement of the breath through the body balances the mind and brain and modulates pain by aligning the perception of challenging (*dukha*) or pleasurable (*sukha*) sensations in the body.

ET is expressed in the spatiotemporal movement of body sensations over time. For [B], “many things come under [our reactions to feelings, such as] *raga*, like hatred is there, anger is there, passion is there, fear is there, fear of something. All these are the diseases according to this text and they're saying that these are the real diseases.” In other words, the arising of reactions (e.g., love, *raga*, or hatred/aversion, *dvesha*) to specific feelings of pleasure (*sukha*) and pain (*dukha*) as perceived in the body in response to CP are also associated with time and associated with CP pathophysiology. As [B] noted, the very first *Vaidya*, “hasn't enlisted any physical disease [diagnostic conditions], actually. They haven't enlisted fever there, they haven't enlisted diarrhea, they haven't enlisted skin diseases … We can see that all the physical diseases basically arise most of the times from the mind, psychological effects.” [B] emphasizes the cause of CP as the arising of the reaction (through aversion, *dvesha*, or love, *raga*) to the emotional or thought sensation in the body (*vedana* or *sukha*) that in turn gives rise to an imbalance in the body that manifests as physical conditions or disease. As [B] notes, “if we see in *Charaka Samhita*, that is another classical text of Ayurveda, the various diseases like fever, [d]iarrhea, [skin] diseases … they are telling [us] that the very first appearance of these diseases in the … ancient times [was] due to some psychological things related to mind.” Thus, the arising of reactions (*raga*, *dvesha*) in the mind to the spatiotemporal movement of embodied sensations (of *dukh*a and *sukha*) over time is associated with the pathophysiology of CP.

ET is expressed through the consciousness of the individual expressed through the *chakras*. The *marma* massage practice connects the consciousness components of the body (*chakras*) with spatiotemporal movement of breath and body sensations in ways that function to balance and rejuvenate. Pain treatment through practices such as *marma* and *panchakarma* in the Ayurvedic physician's description is based on the evaluation and alignment of breath in everyday practices and norms that are manifested through the actions of the body:

“I will advise them a proper diet, then proper nutrition. Then I will advise them to have a regular exercise, proper implementation of dinacharya, after *that ritucharya, ritucharya* is nothing but the fine-tuning of *dinacharya* with the nature. So, you have to make certain changes according to the outside climate. So that will help the patient for healthy living. Then I will suggest them to have some type of herbal teas on regular basis. Then I will suggest them the regular exercise, yoga, *pranayama* along with some massage, even massages mentioned in *dinacharya*, to reduce the dryness of the body … Also, I will suggest my patient to have the *rasayana* treatment, which is a good treatment for rejuvenation. Then also I will suggest them to have the *marma* therapy treatment or *marma* massage, which can be done at home level.” [H]

The *marma* massage may include practices such as *abhyanga* and *svedana* (oil therapy, *taila* massage). As [I] notes, “in *panchakarma*, we use an oil. Suppose we can, for daily application also, if there is pain like in, if there is ligament tear, or if there is muscle tear, and there is persistent pain.” The treatment is tailored to the unique *dosha* composition of each individual. According to [H], “*marma* is the junction between the physiology and consciousness … each major *marma* point corresponds to several *chakras* or energy centers of the body. And when we stimulate these *marma* points, the energy which is clogged is released and it increases the circulation and helps to reduce the pain.” Thus, *marma* practices are employed with the goal to remove the consciousness blocks and help modulate the embodied reaction to pain sensations (*raga* and *dvesha*) over time.

In *panchakarma*, one of the central treatment pathways in Ayurveda, according to [G], “the mind is very much involved … we rejuvenate that mind, or we calm that mind [using] a medicinal purgation for the vitiated [*dosha*].” Similarly, as [G] noted, practices such as yoga also cultivate the consciousness: “while doing *yogasana* [such as the] sun salutation is a kind of concentration or is a kind of thinking of ourselves. *Pranayama* is also thinking of ourselves. Any kind of yoga is also thinking of ourselves … When we are doing sun salutation, their other typical *asana*s with the control on our respiration. While doing such kind of *asana*, we control our mind.” The energy facets of physical movement as defined in yoga align the energy flow with the thought and connect with the energy flow of nature, the environment, and the cosmos. For instance, as [G] noted, there is a connection between these yoga and *marma* practices with food, as described in the case of treatment for knee pain, “so, when we are treating the knee joint, with the help of any kind of medicines or *panchakarma* process, I must, or a physician must look after the … mental status of that person … or any kind of disorder or digestive problem.” Food ultimately gets converted into matter, thought, and energy. The spatiotemporal movement of these shapes the individual experience of CP. Spatiotemporality is conceptualized in ET as an awareness of the relationality of inner time and space with the emotional, cognitive, and consciousness processes through their movement in the body as breath, as body sensations, and as shifts in consciousness (Figure 2).

## Discussion

The Ayurvedic protocols of pain management conceptualize temporality in CP as present moment awareness and as embodied time. PMA comprises a spatiotemporal awareness of cognitive temporal movement, *dosha* operations, and their pathophysiological expression as constructing the body. PMA is cultivated through mind–body practices that support spatiotemporal awareness of thought and sound (e.g., in chants, such as *mantra*s), constituting the individual's cognitive, pathophysiologically experienced environment (Figure 1 and Table 3). ET comprises a spatiotemporal awareness of the relationality of breath, body sensations, and consciousness as constructing the individual's experiential environment. ET is cultivated through practices that produce a spatiotemporal awareness of breath, body, and consciousness (e.g., in *pranayama* yoga, *marma*, and *panchakarma*), constituting the individual's ecosystemic, seasonal, and circadian environment (Figure 2 and Table 3).

PMA is described as cultivating a spatiotemporal awareness of cognitive temporal movement (e.g., thoughts), *dosha* operations (e.g., movement of the three different forms of energy, *vata*, *pitta*, and *kapha*), and their pathophysiological expressions (e.g., pain duration and intensity). PMA is centered in the spatiotemporal equilibrium among these interrelated components (e.g., among the three *doshas* and their expression in body sensations). For instance, as the *doshas* manage the functioning of the body, a state of dynamic equilibrium will support the elimination of toxicity, alignment with cognitive temporal movement (governed by specific *dosha* functions), and regulation of body sensations. The spatiotemporality of cognitive movement expressed as thought is regulated through practices that center the body and mind (e.g., through particular forms of sound, as in *mantra*s). Thus, the Ayurvedic physician will examine the relationality of the *dosha* processes governing the operations of the body and the mind. The spatiotemporal practices in PMA will focus on individualized centering and balancing functions such as those emphasizing positive and resilient qualities using *mantras,* yoga *asanas,* and *pranayama* (Figure 1 and Table 3).

ET is described as cultivating a spatiotemporal awareness of movement of breath (e.g., as *prana*), of the body's emotional and psychosocial sensations (e.g., of pleasure and pain), of the reactions to body sensations (e.g., of positive and negative affect to feelings of pain or pleasure), and in their intersection with consciousness (e.g., as *chakras*). ET is centered in the spatiotemporal expression of energy, body sensations, psychosocial reactions, and consciousness in an experiential ecosystemic environment. For instance, embodiment references the integrated experience of movement of breath through the body, the movement of body sensations, and of nature and the ecosystemic environment in consciousness. The spatiotemporality of breath, of body sensations and their reactions, and consciousness is expressed through practices that center the integration of the body, its perceptual mechanisms, and the consciousness (e.g., as in *marma* massage). Thus, the Ayurvedic physician will examine the spatiotemporal nature of the emotional, cognitive, and consciousness processes. The spatiotemporal practices in ET will emphasize rejuvenation and self-reflexivity using *marma, panchakarma, pranayama* yoga, and medicinal herbs (Figure 2 and Table 3).

The study findings present an experiential and relational framework conceptualizing temporality through its spatial dimensions as an organizing principle in CP management. While temporality focuses on the representation of experiences and relations over time, spatiotemporality emphasizes a constructionist approach by centering balance and equilibrium in the passage of time, consciousness, and subjective experience of the body in relationship with its environment. Spatiotemporality highlights pain as a materially grounded experience that evokes cognition, emotion, perception, and consciousness body-in-context. Research shows that CP disrupts the experience of lived temporality (25) CP cognition, and conscious experiencing of sensations through cognitive processing (36). The body dissociation associated with CP is centered through spatiotemporality as PMA and ET in embodied, relational, and body-aware ways. Biopsychosocial approaches to CP management emphasize mindfulness meditation and cognitive therapy as modes for altering pain catastrophizing (e.g., by altering cognitive content, processing, and negative affectivity) or restructuring pain-related cognitive content, making adaptive changes necessary to counter pain perception (78). The spatiotemporal frame balances the emphasis on cognition in Mindfulness-based cognitive therapy (MBCT)-based approaches through the integration of the PMA and ET dimensions. The spatiotemporality of PMA and ET cultivates an experiential and emergent quality that has the potential to extend tailored multimodal treatment plans that comprise pain neuroscience education, cognition-targeted exercise therapy, sleep and stress management, and/or dietary interventions to support CP management. Integrating the ontological–epistemological framework of Ayurvedic whole medicine attends to the spatiotemporal environment in nuanced ways to cultivate a dialectical relationship with the reflexive self in PMA [e.g., self-awareness (35)] and the embodied internal–external temporal environment referenced by biographical–historical, geographical–universal environments in ET.

Understanding time perception as a component of an individual's psychological phenomena (79), PMA in CP management emphasizes the alignment of recollections and anticipation with the spatiotemporal awareness of the body and change (of sensations) in the present, past, and future. Integrating cognitive temporal movement and *dosha* awareness extends current research examining temporality in multisensory integration and self-awareness (9, 16, 17) as they contribute to body ownership and acceptance in CP management (15) by expanding the conceptualization of temporality from the subjective representation of experiences and relations over time, time perceptions, and flow experiences (53). PMA introduces spatiotemporality as a state of dynamic equilibrium cultivating an awareness of internal time, external duration, and the self with subjective experience (21) through centering the spatiotemporal frame in the pathophysiologically experienced environment. Extending temporal constructs such as the conceptualization of the passage of time (21, 22) through the lens of spatiotemporality centers the internal and external environment of the body in its lived space and attends to the disruption in lived temporality experienced by CP patients. Time is assessed as a component of spatiotemporality in clinical CP diagnosis, treatment, and evaluation when the Ayurvedic physician examines the patients' daily and seasonal lifestyle, mind, and emotions as an interconnected whole in PMA and ET. Assessing the experience of longitudinal time through PMA and ET helps the Ayurvedic physician focus integratively and in environmentally centered and embodied ways on the subjectively experienced past, present, and future by focusing on sensemaking related with past choices (43), prioritizing what is meaningful in the moment (80), or imagining a purposeful future (81).

The cultivation of PMA can support the disruption of lived temporality and the experience of time (25) in CP through the practices employed by the Ayurvedic physician. The Ayurvedic physician attends to the relationship of internal time with spatiotemporality through sound and the body's energy balance as expressed through daily practices and the lived environment. Mindfulness practices can be enhanced through PMA by conceptualizing being present in the moment with awareness of thoughts, the pathologies of the body, and the doshas in relationship with the spaces of one's lived environment and the natural world. Similarly, the awareness of spatiotemporality in daily practices through sound (*mantra*s) extends the understandings of mental health in relationship with cognitive activity as a form of temporal and referencing the spatiotemporal nature of the relations between the mind and emotions. Self-reflexivity supports the provider–patient relationship (5) in PMA as cultivating an awareness of the relationality of cognitive movement, *dosha* operations, and their pathophysiological expression in the body. PMA processes align cognition and pain processing to highlight how enjoining awareness of the relationality of space with temporality advance techniques incorporating dialectic integration of change with acceptance of the present.

Grounding temporality with spatial awareness in ET connects intentionality with action upon pain duration, future time perspective, and subjective perception of time (37). ET deepens the conceptualization of space to include internal bodily spaces, the lived environment, and the natural environment that shapes the body's functions to cultivate an awareness of spatiotemporality as integrated with the embodied sensory and psychosocial processes of the body. The Ayurvedic assessments of *dosha* analysis, tongue analysis, and its relationship with the *doshas* to assess temporal change are key to conceptualizing the embodied mind–body relationship. Integrating spatial awareness in ET attends to the intentional recollection and spatiotemporal (re)situatedness of sensory memories, emotions, and experiences (41). Spatiotemporality of embodied processes such as those of flow of breath (through *pranayama*) and the body's energy centers (through *chakra* meditation or *marma* massage) enhances awareness of how pain perceptions are constructed through continual interaction with bodily, lived, and environmental spaces. Spatiotemporality of ET processes deepens conceptualization of unified flow experiences (29, 38, 41) in adaptive mutual engagement with pain experiences and cultivate ways of conceptualizing spaces as temporally constructed in CP management.

The conceptual explication of spatiotemporality as ET extends the understandings of how the pathophysiological, behavioral, and emotional processes of CP relate with temporal dynamics (60) and expansion in the perception of subjective time and space (82). Conceptualizing spatiotemporality as ET expands how CIM provider co-engagement in CP management (83) is constituted as perceptual subjective process over time through cultivating awareness of intentionality in movement of breath, thoughts, and emotions that inform the bi-directional relationship between cognition and pain and the disruption of cognitive processing in CP (34, 36). Emerging perspectives such as the ecological momentary assessment approach assessing the modulation of pain experiences over time (61) in relationship with their natural daily environments can be extended through an embodied understanding of their relationship with the patient's daily activities and circadian variability (84). Examining the qualities of bodily vitality (as *ojas*), strength (as *bala*), and type (as *prakriti*) through relations of space and time in alignment with circadian and seasonal temporal cycles offers an expansive and integrated frame for conceptualizing spatiotemporal relationships of the mind and body.

The study is limited by its very small, expert-focused sample size in a complex knowledge domain spanning temporality, Ayurvedic medicine, and biopsychosocial CP management. The generalizability of the study is further limited by the fact that it was designed and conducted by a single researcher. Future studies can explicate the clinical contribution of constructs such as temporality and belief (85) that are significant components of Ayurvedic medical ontologies and respond to the call to enhance specialized clinical practice in Ayurveda (86) (see also Appendix 2). The process of observing change over a spatiotemporal frame, such as through the moment-by-moment movement of the breath in PMA and ET supports both the expression and perception of CP. The Ayurvedic physician employs their knowledge of individual operation of *doshas,* bodily vitality (as *ojas*), bodily strength (as *bala*)*,* integral individual bodily nature (as *prakriti*), and ET (as *kaal*) to recommend multimodal approaches comprising nutrition, physiological postures through yoga and massage, movement through practices involving physiological purification (e.g., *nasya)*, and a whole-person approach through the central Ayurvedic program of *panchakarma* and related modalities and therapies. In centering patient empowerment, the Ayurvedic physician incorporates an assessment of the patient's mental and spiritual strength (*bala*) in the treatment of pain in alignment with the daily and seasonal temporal cycles.

The findings address a gap in the conceptualization of temporality in CP management and offer an ontologically diverse conceptualization of spatiotemporality in envisioning how space and temporal relations can be conceptualized in integrative CP models (87–89). Incorporating PMA and ET in adaptive relationship with the lived environment, cognitive and emotional processes, and circadian and seasonal changes enhances how clinical approaches to CP management can consider pain subtype, structural pathology, and patient biopsychosocial profile by attending to the relations of space and temporality in nuanced and complex ways.

## Author's note

An earlier version of this paper was presented virtually at the International Communication Association Annual Convention, and an additional version has been previously presented virtually at the National Communication Association Annual Convention. Previous versions have also been presented as an abstract at the Global Approaches of Integrative Oncology session of the National Cancer Institute and the Trans NCI-NIH Conference on International Perspectives on Integrative Medicine for Cancer Prevention and Cancer Patient Management (virtual poster presentation). This study presents part of the larger data set. Additional publications from this data set can be found under References, Agarwal, 2020.

## Data Availability

The raw data supporting the conclusions of this article will be made available by the author, without undue reservation.

## Appendix 1

### Selected salient conceptual domains and constructs explored in the semi-structured interview

#### Protocol

• Participant information: Name/Age/Qualifications (specialization)/Degree credentials/Profession/How many years in current profession and position/Location
• Domain: Chronic pain management, Ayurvedic principles and practices, connection of *ahar/dravya/shastra*/with *chikitsa* focus on connection of *manas/atman/shareer* in Ayurvedic practices and their effectiveness (how described, perceived, conceptualized)
• Understanding tailoring principles in practice for pain management mind–body integrative approach (*dosha/panchmahabhoota/rasa/guna*)
• Understanding physician communication of abstract concepts and implementation in practice by patient
• Evolution to incorporate role of *desha* and environment from *Charaka Samhita* to present-day.

#### Semi-structured interview protocol domains

I. Mind–body practice protocol: Mind and body in relationship with each other
II. Communication of mind–body–spirit practices for CP
III. CIM practice protocol
IV. Focusing attention (*man*) and regulating emotions in mind–body practices:
V. CIM practices associated with pain management
VI. How does the Ayurvedic physician help the patient address the following pain aspects in their lives? (Fatigue/Stress/Fear/Quality of life/Cognitive impairment/Body distress)
VII. Understanding how Ayurveda communicates the relationship between healing, functionality, quality of life, and wellbeing in CP management

## Appendix 2

### Ayurvedic terminology in clinical practice and interpretive premise of study

The interpretive premise of the study implies that the thematic findings reference the author's interpretive lens to the Ayurvedic ontological and philosophical thought as reflected in the themes in the Results section of the study. Accordingly, the interpretive frames identified in this exploratory study are not clinical findings intended to reflect the medical training informing Ayurvedic physicians' clinical practice protocols as applied in Ayurvedic and integrative medicine in India. Rather, they reflect the author's identification of spatiotemporality as a philosophical and interpretive construct that may be further evaluated, examined, and explicated in future studies to enrich its dimensionality in practice through a diverse knowledge translation provided by the findings in this study. To this end, the study provides one instance of an effort to provide one instance of a complex translation of the knowledge base and study field of the examination of Ayurvedic philosophy from a diverse lens.

For related research findings specifically from a clinical practice perspective, for instance, Ayurvedic physicians will assess temporality in a highly complex and nuanced manner to examine when pain arises or peaks (i.e., pain variation), its relationship with diurnal and seasonal temporality, age, geography, diet, and lifestyle, among a range of variables. Ayurvedic physicians will also examine the specific dominance of the dosha function with each of these variables (e.g., the gradual decline of *kapha dosha* as the person ages and its relationship with pain variation). Similarly, a range of principles and precepts is employed to inform such assessments of health and disease (e.g., the principle of hot, *ushna*, and cold, *sheeta*) and their related pathogenesis features (90). Greater clarity in such constructs can help tailor Ayurvedic treatments to patient priorities for choosing Ayurveda treatment [e.g., potential to eradicate disease, belief, direct and indirect evidence of efficacy and safety in managing the clinical condition of interest (91)].
